# Non-Phosphorylatable PEA-15 Sensitises SKOV-3 Ovarian Cancer Cells to Cisplatin

**DOI:** 10.3390/cells9020515

**Published:** 2020-02-24

**Authors:** Shahana Dilruba, Alessia Grondana, Anke C. Schiedel, Naoto T. Ueno, Chandra Bartholomeusz, Jindrich Cinatl Jr, Katie-May McLaughlin, Mark N. Wass, Martin Michaelis, Ganna V. Kalayda

**Affiliations:** 1 Department of Clinical Pharmacy, Institute of Pharmacy, University of Bonn, 53121 Boon, Germany; 2 Department of Pharmaceutical and Medicinal Chemistry, Institute of Pharmacy, University of Bonn, 53121 Boon, Germany; 3 Section of Translational Breast Cancer Research, Department of Breast Medical Oncology, The University of Texas MD Anderson Cancer Center, Houston, TX 77230-1439, USA; 4 Institute of Medical Virology, Goethe University Hospital Frankfurt, 60590 Frankfurt/Main, Germany; 5 Industrial Biotechnology Centre and School of Biosciences, School of Biosciences, University of Kent, Canterbury CT2 7NJ, UK

**Keywords:** PEA-15, ovarian cancer, cisplatin sensitivity

## Abstract

The efficacy of cisplatin-based chemotherapy in ovarian cancer is often limited by the development of drug resistance. In most ovarian cancer cells, cisplatin activates extracellular signal-regulated kinase1/2 (ERK1/2) signalling. Phosphoprotein enriched in astrocytes (PEA-15) is a ubiquitously expressed protein, capable of sequestering ERK1/2 in the cytoplasm and inhibiting cell proliferation. This and other functions of PEA-15 are regulated by its phosphorylation status. In this study, the relevance of PEA-15 phosphorylation state for cisplatin sensitivity of ovarian carcinoma cells was examined. The results of MTT-assays indicated that overexpression of PEA-15AA (a non-phosphorylatable variant) sensitised SKOV-3 cells to cisplatin. Phosphomimetic PEA-15DD did not affect cell sensitivity to the drug. While PEA-15DD facilitates nuclear translocation of activated ERK1/2, PEA-15AA acts to sequester the kinase in the cytoplasm as shown by Western blot. Microarray data indicated deregulation of thirteen genes in PEA-15AA-transfected cells compared to non-transfected or PEA-15DD-transfected variants. Data derived from The Cancer Genome Atlas (TCGA) showed that the expression of seven of these genes including *EGR1* (early growth response protein 1) and *FLNA* (filamin A) significantly correlated with the therapy outcome in cisplatin-treated cancer patients. Further analysis indicated the relevance of nuclear factor erythroid 2-related factor 2/antioxidant response element (Nrf2/ARE) signalling for the favourable effect of PEA-15AA on cisplatin sensitivity. The results warrant further evaluation of the PEA-15 phosphorylation status as a potential candidate biomarker of response to cisplatin-based chemotherapy.

## 1. Introduction

Platinum-based drugs have been used to treat ovarian cancer since the late 1970s and cisplatin, followed by carboplatin-based combinations, has been the standard of care for over 15 years. As most patients relapse and ultimately succumb to ovarian cancer, new strategies are urgently required to improve survival. Cisplatin is believed to exert its cytotoxic effects via its interaction with DNA and formation of DNA adducts, primarily intrastrand crosslinks. This initiates signal transduction pathways involving among others Ataxia Telangiectasia Mutated (ATM) protein, p53, p73 and mitogen-activated protein kinases (MAPK), eventually resulting in cancer cell apoptosis [1]. However, DNA damage-mediated apoptotic signals can be diminished leading to the development of resistance, which represents a major restraint of cisplatin-based chemotherapy [2].

Among the constituents of DNA damage signalling, mitogen-activated protein kinases (MAPKs) are of particular interest for their diverse functions in modulating cell death machineries. MAPKs are a family of structurally-related serine/threonine protein kinases that coordinate various extracellular stimuli to regulate cell growth and survival [3,4,5]. There are three major subfamilies of MAPKs: extracellular signal-regulated kinase (ERK1/2), stress-activated protein kinase (SAPK)/c-Jun N-terminal kinase (JNK) and p38 MAPK [2].

ERK1 and ERK2 are homologous isoforms that share the same substrate specificity in vitro [6]. These 44- and 42-kDa proteins that phosphorylate a multitude of protein substrates [7,8] share 85% of amino acid identity. In resting conditions, ERK1/2 is anchored in the cytoplasm by its association with MEK1/2 [9], the microtubule network [10] or with phosphatases, which contain a nuclear export signal (NES) [11]. Upon stimulation, ERK1/2 becomes phosphorylated at threonine and tyrosine residues resulting in its separation from MEK1/2. Then ERK1/2 translocates into the nucleus by passive diffusion of the monomer [12], active transport of the dimer [13] or by a direct interaction of ERK1/2 with the nuclear pore complex [14]. Upon translocation, activated ERK1/2 phosphorylates the ternary complex factors Elk-1, Sap-1a and TIF-IA (growth dependent transcription initiation factor) [15,16,17]. Phosphorylation of Elk-1 enhances transcription of growth-related proteins, such as c-Fos [18].

Phosphoprotein enriched in astrocytes -15 kDa (PEA-15) is a small scaffold protein, ubiquitously expressed and highly conserved among mammals [19,20,21]. It is involved in the regulation of several cellular functions, including glucose metabolism, cell proliferation, apoptosis and survival [22]. Its expression has been shown to be elevated in tumours, including human glioma and mammary carcinomas [23,24], and in cell lines derived from human larynx, cervix and skin tumours [24,25]. PEA-15 may function as a tumour promoter or suppressor, regulating both proliferation and apoptosis [26].

PEA-15 has two phosphorylation sites at Ser104 and Ser116 and is preferentially phosphorylated by protein kinase C (PKC) at Ser104 and by calcium/calmodulin-dependent protein kinase II (CaMKII) or Akt at Ser116 [20,27,28]. PEA-15 can bind both ERK1/2 and phosphorylated ERK1/2 (p-ERK1/2) with equal affinity [29]. PEA-15 phosphorylation releases ERK1/2 resulting in its translocation to the nucleus and activation of the nuclear transcription factor Elk-1 as well as other transcription factors promoting cell proliferation. As PEA-15 has a nuclear export sequence, it is almost exclusively confined to the cytoplasm [30]. PEA-15-mediated cytoplasmic sequestration of ERK1/2 was reported to suppress tumourigenicity in ovarian cancer by diminishing the activity of Elk-1 [31]. Another study found that PEA-15 expression inhibited cell proliferation by autophagy involving ERK1/2 activation [32]. The functions of PEA-15 are tightly regulated by its phosphorylation status [22]. The impact of PEA-15 phosphorylation was investigated by Lee et al. [33] in ovarian cancer tissue samples revealing that tissues from high-grade ovarian tumour were significantly more likely than adjacent normal tissues to express PEA-15 phosphorylated at both sites. The authors used phosphomimetic and non-phosphorylatable PEA-15 mutants where the two serine residues of PEA-15 at 104 and 116 positions were either replaced by two aspartic acid (PEA-15DD) or two alanine (PEA-15AA) residues, respectively. The non-phosphorylatable PEA-15AA exerted a more pronounced antitumourigenic effect in ovarian cancer than did the phosphomimetic PEA-15DD [33]. To study the role of PEA-15 phosphorylation in paclitaxel sensitivity in ovarian cancer, PEA-15AA and PEA-15DD were overexpressed in SKOV-3.ip1, OVTOKO and HEY cells. All three cell lines showed enhanced sensitivity to paclitaxel when phosphomimetic PEA-15DD was overexpressed, while nonphosphorylatable PEA-15AA augmented resistance to paclitaxel [34].

The aim of this work was to investigate the influence of PEA-15 and its phosphorylation status on cisplatin sensitivity in ovarian carcinoma cells. We show that the non-phosphorylatable PEA-15AA sensitises ovarian cancer cells to cisplatin. The results warrant further evaluation of the phosphorylation state of PEA-15 in order to consider it as a potential biomarker of tumour sensitivity to cisplatin.

## 2. Materials and Methods

### 2.1. Chemicals

3-(4,5-dimethylthiazol-2-yl)-2,5-diphenyltetrazolium bromide (MTT) was purchased from AppliChem (Darmstadt, Germany), dimethylsulfoxide (DMSO) was ordered from Riedel-de Haën, (Seelze, Germany), bovine serum albumin (BSA), all-trans-retinoic acid and cisplatin were obtained from Sigma-Aldrich (Steinheim, Germany), foetal calf serum, penicillin-streptomycin solution, IMDM^TM^ Medium, trypsin-EDTA solution were ordered from PAN^TM^ Biotech (Aidenbach, Germany) and ultrapure water was obtained using a Purelab Plus ^TM^ system from ELGA Labwater (Celle, Germany).

### 2.2. Cell Lines and Cell Culture

The SKOV-3 ovarian carcinoma cell line (ATCC^®^ HTB77^TM^) was from American Type Culture Collection (ATCC). The cisplatin-resistant ovarian carcinoma cell line EFO27^r^CDDP^2000^ was derived from the Resistant Cancer Cell Line (RCCL) collection (www.kent.ac.uk/stms/cmp/RCCL/RCCLabout.html). Cell backups were frozen with 10% DMSO. Cells were grown as monolayers in IMDM^TM^ medium supplemented with 10% foetal calf serum, 100 IU/mL penicillin, 0.1 mg/mL streptomycin in a humidified atmosphere containing 5% CO_2_. The medium of EFO27^r^CDDP^2000^ cells was supplemented with 2 µg/mL cisplatin. Every ten passages a new backup of cells was thawed in order to avoid alterations in cell features during cultivation.

### 2.3. Plasmid Transfection

Cells were transfected with plasmids of interest using K2^®^ transfection system (Biontex Laboratories GmbH, Munich, Germany) according to the manufacturer’s instruction. Plasmids used in this study include three DNA constructs of pcDNA3-HAR36 containing two mutated PEA-15 protein versions and the empty vector. These plasmids were kindly provided by Prof. Naoto T. Ueno (Breast Medical Oncology, MD Anderson Cancer Center, University of Texas, Houston, TX, USA). The constructs were originally made by Ramos et al. [21]. The two mutated versions of PEA-15 protein were PEA-15AA, in which two serine residues at 104 and 116 positions were replaced with two alanine (A) residues, and PEA-15DD, in which the same serine residues were replaced with aspartic acid (D). Cells were used for protein expression and chemosensitivity analysis 48 h after transfection.

### 2.4. Cell Fractionation

Nuclear/cytosol fractionation kit (Biovision Inc., Milpitas, CA, USA) was used for separating the cytosolic and the nuclear fractions of the transfected cells according to the manufacturer’s protocol. All steps of the fractionation were performed on ice. To analyse the efficiency of the fractionation, Western blot analysis was performed for each fraction (nuclear or cytosolic) as described below. As a marker for the cytosolic fraction, GAPDH was used. For the nuclear fraction, Lamin B1 was used as an indicator of the purity of the nuclear fraction. No cross-contamination was assumed when the indicator was detected only in the expected fraction.

### 2.5. Western Blot

Cells were fractionated as mentioned above or lysed in lysis buffer (50 mM Tris-HCl, 150 mM NaCl, 0.1% SDS, 1 mM NaF, 2 mM Na_3_VO_4_, 70% NP-40, 0.5% sodium deoxycholate, 6.24 mM benzamidine and 0.5 mM PMSF, all from Sigma-Aldrich, Steinheim, Germany) for 30 min on ice. Then the cells were sonicated on ice using a sonicator with the settings: 60% power, 5 s pulse, 30 s interval, 3 pulses per sample. The suspensions were centrifuged for 5 min at 18,620× *g* at 4 °C, and the protein content in the supernatants was measured using the bicinchoninic acid assay (BCA^TM^ Protein Assay Kit, Pierce, Rockford, IL, USA) [35]. Samples containing 30 μg total protein were subjected to electrophoresis in 12% SDS-polyacrylamide gel and transferred to a PVDF membrane (Carl Roth GmbH, Karlsruhe, Germany). The membranes were blocked in 5% milk powder in TBS-T (0.2% Tween-20) for 1 h, rinsed three times with TBS-T and incubated at 4 °C overnight with primary antibodies diluted in TBS-T with 5% BSA. After washing three times with TBS-T, incubation with the secondary antibody diluted 1:1000 in TBS-T with 5% milk powder for 1.5 h followed. The monoclonal mouse antibody against UGT1A (diluted 1:1000) sc-271268, the polyclonal rabbit antibody against Nrf2 (diluted 1:500) sc-722 were from Santa Cruz Biotechnology, Heidelberg, Germany, the monoclonal mouse antibody against HA MMS-101-P (diluted 1:500) was received from Covance Inc., PA, USA. The secondary HRP-conjugated goat anti-rabbit (diluted 1:1000) SBA-4030-05 was obtained from Biozol Diagnostica Vertrieb GmbH, Eching, Germany, the rabbit polyclonal antibody against p-ERK1/2 (Thr202/Tyr204) 9101 (diluted 1:1000) was ordered from Cell Signaling Technology Europe B.V., Frankfurt (Main), Germany, and the Peroxidase AffiniPure goat anti-mouse (diluted 1:5000) 115-035-003 was from Jackson ImmunoResearch Europe Ltd., Cambridgeshire, UK. The detection was performed using a Molecular Imager ChemiDoc^TM^ XRS^+^ System from Bio-Rad Laboratories GmbH, Munich, Germany. After subsequent triple washing with TBS-T, the membranes were incubated for 30 min. with the rabbit antibody against GAPDH (GTX100118, Biozol Diagnostica Vertrieb GmbH, Eching, Germany) diluted 1:20000 or for 1 h with the rabbit polyclonal antibody to Lamin B1 (GTX-103292, Biozol Diagnostica Vertrieb GmbH, Eching, Germany) diluted 1:1000 in TBS-T with 5% BSA. After rinsing with TBS-T, the incubation with the secondary antibody and detection were performed as described above. Densitometric analysis was performed using ImageLab^TM^ 5.1 software (Bio-Rad Laboratories, Hercules, CA, USA).

### 2.6. MTT Assay

Cell sensitivity to cisplatin, retinoic acid or their combination was assessed using an MTT-based assay [36]. In brief, cells were seeded in 96-well microtiter plates (1 × 10^4^ cells/well) and allowed to attach overnight. Then medium was removed and nine subsequent dilutions of retinoic acid or cisplatin in medium were added to the cells in triplicate (100 µL/well). For the combination treatment, cisplatin dilutions each contained 20 µM retinoic acid. After 47 h of incubation, 20 µL of a 5 mg/mL MTT solution in phosphate buffered saline (PBS) was added to each well, and the cells were incubated at 37 °C for 1 h. The supernatant was discarded, and the formazan crystals formed were dissolved in 100 µL DMSO. Absorbance of the dye was measured at 570 nm with background subtraction at 690 nm using a Multiskan Ascent^®^ microtiter plate reader (Thermo Fisher Scientific, Langenselbold, Germany). The results were analysed and the pEC_50_ values (pEC_50_ = -log EC_50_, EC_50_ = half maximal effective concentration) were estimated for each independent experiment with the GraphPad Prism^TM^ 6 analysis software package (GraphPad Software, San Diego, CA, USA) using non-linear regression (sigmoidal dose response, variable slope). The mean pEC_50_ values were calculated from the results of several independent experiments and used for the determination of the respective EC_50_ values.

### 2.7. cDNA Microarray Analysis

SKOV-3 cells were transfected with PEA-15-HA- (empty vector, EV), PEA-15AA- or PEA-15DD-containing plasmids, respectively, as described above. Twenty-four hours after transfection, cells were treated with 15 µM cisplatin (this concentration corresponds to the EC_50_ of cisplatin in EV-transfected cells as measured after 48 h of incubation) for 24 h and then the total RNA of the cells was extracted using my-Budget RNA mini kit following the instructions provided by the manufacturer. Next generation transcriptome-wide gene-level expression profiling using Clariom^TM^ S assay was performed by Life and Brain GmbH (Bonn, Germany). The microarray data have been deposited into the Gene Expression Omnibus database under the accession number GSE144041.

The raw data of the array were collected as CEL files and analysed using the Transcriptome Analysis Console (TAC 4.1, Thermofisher Scientific, Waltham, MA, USA) software. The gene expression was analysed with the Gene Level Signal Space Transformation-Robust Multi-Chip Analysis (SST-RMA) summarization method. Data obtained from the microarray were normalised by the robust multiarray average method [37]. A probe set was considered expressed if ≥50% samples had DABG (Detected Above Background) values below DABG threshold (*p* < 0.05). Statistical significance of the differences in gene expression was analysed using Limma [38]. Differential expression was assumed at a *p* value <0.05 and a fold change in expression ≥2 or ≤−2.

### 2.8. Correlation of Tumour Gene Expression Levels with the Survival of Cisplatin-Treated Patients in The Cancer Genome Atlas (TCGA)

Gene expression data from patient tumours was derived from The Cancer Genome Atlas (TCGA) [39,40] via the GDC Data Portal (https://portal.gdc.cancer.gov). The Bioconductor R package TCGAbiolinks was used to obtain corresponding clinical data. Tumour gene expression data and cisplatin response data were available for 779 patients representing 23 different cancer types (adrenocortical carcinoma, *n* = 2; bladder urothelial carcinoma, *n* = 78; breast invasive carcinoma, *n* = 2; cervical squamous cell carcinoma and endocervical adenocarcinoma, n = 122; cholangiocarcinoma, *n* = 4; lymphoid neoplasm diffuse large b-cell lymphoma, *n* = 2; oesophageal carcinoma, *n* = 14; glioblastoma multiforme, *n* = 7; head and neck squamous cell carcinoma, *n* = 94; kidney renal papillary cell carcinoma, *n* = 1; liver hepatocellular carcinoma, *n* = 4; lung adenocarcinoma, *n* = 84; lung squamous cell carcinoma, *n* = 72; mesothelioma, *n* = 34; ovarian serous cystadenocarcinoma, *n* = 115; pancreatic adenocarcinoma, *n* = 2; sarcoma, *n* = 3; skin cutaneous melanoma, *n* = 9; stomach adenocarcinoma, *n* = 44; testicular germ cell tumours, *n* = 53; thymoma, *n* = 5; uterine corpus endometrial carcinoma, *n* = 21; uterine carcinosarcoma, *n* = 7). Overall survival (OS) was defined as days to last follow-up or death as previously described [41].

Genes whose expression was undetected (FPKM = 0, FPKM – fragments per kilobase of exon model per million reads mapped) in >10% samples were removed from the investigation as previously described [42]. Patients were stratified by cancer type, and the median expression for each gene was calculated. Genes with expression values above the median were considered highly expressed, while those below the median were considered to have low expression. Patients were then re-amalgamated before being stratified by drug treatment for pan-cancer analysis.

Cox proportional hazards regression was used to calculate the hazard ratio for cohorts expressing high (above-median) vs. low (below-median) levels of a given gene. Calculations were performed using the R survminer and survival packages. *p*-values in each case are the result of a log rank (Mantel-Cox) test, which assesses whether there is a significant difference between the survival of two independent groups. Hazard ratios quoted refer to values for ‘low’ (below median) expression for each gene in the model, with values >1 indicating increased hazard (i.e., reduced OS) and values <1 indicating decreased hazard (i.e., increased OS). Multiple test correction was again performed for each of the samples (Benjamini-Hochberg method [43]). The false discovery rate (FDR) values are thresholds that indicate whether *p*-values are below or above the respective significance levels.

### 2.9. Statistical Analysis

GraphPad Prism^TM^ 6 was used for analysing and plotting the data. Protein expression data were compared using one-way ANOVA with a Holm-Sidak post-test. Unpaired *t*-test was used to compare pEC_50_ values. *p* values of <0.05 were considered significant.

## 3. Results

### 3.1. PEA-15AA Sensitised SKOV-3 Cells to Cisplatin

**Figure 1:**
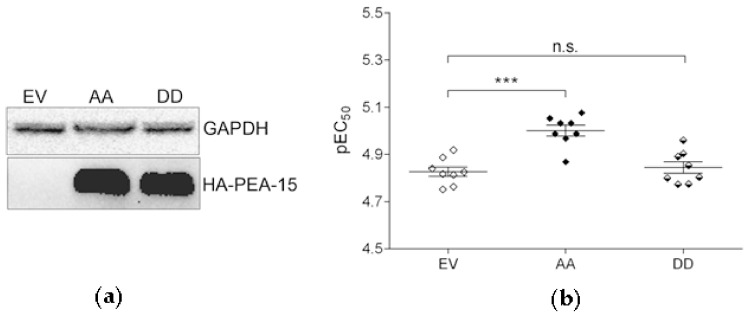
(**a**) Expression of hemagglutinin (HA)-tagged phosphoprotein enriched in astrocytes (PEA-15) in SKOV-3 cells after transfection with the HA-tagged empty vector (EV), PEA-15AA (AA) and PEA-15DD (DD). GAPDH was used as a loading control. (**b**) Cisplatin sensitivity (pEC_50_, mean ± SEM, *n* = 8) of transfected SKOV-3-EV (EV), SKOV-3-AA (AA) and SKOV-3-DD (DD) cells. *** *p* < 0.001, n.s. = not significant.

In order to assess if the phosphorylation status of PEA-15 affects cisplatin sensitivity, the non-phosphorylatable PEA-15AA (further AA) and the phosphomimetic PEA-15DD (further DD) were overexpressed in SKOV-3 cells by liposome-mediated transient transfection. Control cells were transfected with the empty vector (further EV). The efficiency of the transfection was confirmed by Western blot (Figure 1a). PEA-15AA overexpression significantly increased the sensitivity of SKOV-3 cells to cisplatin compared to the empty vector-transfected cells (Figure 1b). This was not the case upon PEA-15DD overexpression. The EC_50_ in SKOV-3-AA cells was lower (EC_50_ = 9.9 µM) than in SKOV-3-EV cells (14.9 µM), while SKOV-3-DD cells (EC_50_ = 14.3 µM) showed similar cisplatin sensitivity as the SKOV-3-EV controls.

In addition, we confirmed the effect of PEA-15AA on cisplatin sensitivity in the cisplatin-resistant EFO27^r^CDDP^2000^ ovarian carcinoma cell line. Non-phosphorylatable PEA-15 significantly sensitised EFO27^r^CDDP^2000^ cells to the platinum drug (Figure S1). The EC_50_ value after PEA-15AA transfection was 25.1 µM and thus lower than that of 33.1 µM in cells transfected with the empty vector. Overexpression of PEA-15DD did not have any significant effect (EC_50_ = 30.9 µM) compared to the empty-vector control.

### 3.2. PEA-15AA-Transfected SKOV-3 Cells Contain more Cytosolic p-ERK1/2 than PEA-15DD-Transfected Cells

**Figure 2:**
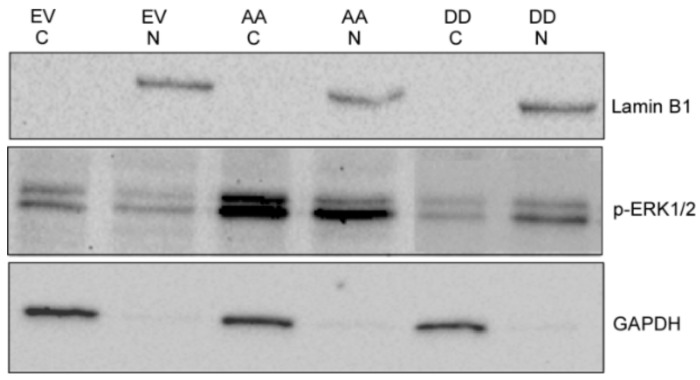
A representative Western blot of phosphorylated extracellular signal-regulated kinase1/2 (p-ERK1/2) expression in nuclear and cytosolic fractions of the SKOV-3 cells transfected with empty vector (EV), PEA-15AA (AA) and PEA-15DD (DD). GAPDH and Lamin B1 were used as the markers and loading controls of cytosolic (C) and nuclear fractions (N), respectively.

EV-, PEA-15AA- and PEA-15DD-transfected SKOV-3 cells were fractionated to separate the nuclear and cytosolic fractions. Figure 2 presents relative expression of activated ERK in the cytosolic and nuclear fractions of the same samples following transfection with different mutants of PEA-15, loaded on the same gel. In SKOV-3-AA cells, the cytosolic fraction contained more p-ERK1/2 than the corresponding nuclear fraction of the same cell lysate, while in SKOV-3-DD cells the opposite was observed (Figure 2). This provides a proof of concept that PEA-15 unphosphorylated at both Ser104 and Ser116 (PEA-15AA) retains p-ERK1/2 in the cytoplasm of SKOV-3-AA cells while the phosphomimetic PEA-15DD does not keep p-ERK1/2 in the cytoplasm, promoting an increase in p-ERK1/2 nuclear accumulation in SKOV-3-DD cells.

### 3.3. Differentially Expressed Genes in Transfected Cells

**Figure 3:**
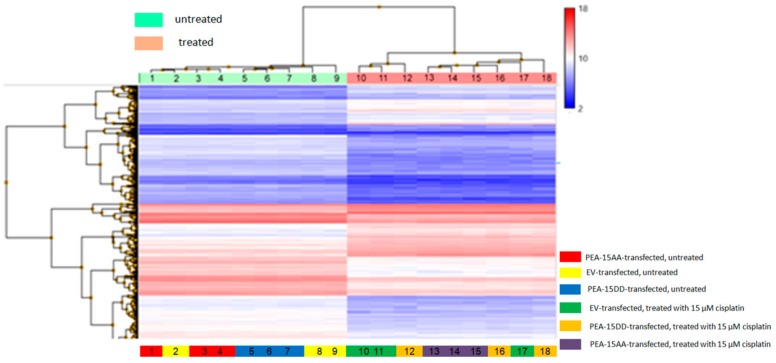
Heatmap of the transcriptome-wide Clariom^TM^ S array, regulated genes with fold change cut-off at 2.0 for differentially expressed genes and a *p*-value cut-off at 0.05 are shown.

As PEA-15 has many other functions inside the cells beside controlling ERK1/2 localisation, a microarray analysis was warranted to identify genes, which may account for enhanced cisplatin sensitivity in PEA-15AA-transfected cells. Clariom^TM^ S assay was performed to analyse the differences in gene expression in untreated and cisplatin-treated SKOV-3-EV, SKOV-3-AA and SKOV-3-DD cells. Genes were considered to be differentially expressed when the fold change was ≥2 and the *p* value was <0.05. The resulting heat map of regulated genes shows clear clustering between treatment conditions (Figure 3).

Table 1 shows the number of differentially expressed genes in EV-, PEA-15AA- and PEA-15DD-transfected cells after cisplatin exposure and in untreated transfected cells. Prior to cisplatin exposure, only three genes were differentially regulated between untreated SKOV-3-EV and untreated SKOV-3-AA. Between SKOV-3-EV and SKOV-3-DD, the number of differentially expressed genes was 18, while for SKOV-3-AA and SKOV-3-DD the number was 10. Following cisplatin treatment, 4430 genes were differentially regulated in EV-transfected cells, while in PEA-15AA- and PEA-15DD-transfected cells the numbers were 4196 and 4110, respectively.

### 3.4. Correlation of Genes Differentially Expressed in the Comparisons Untreated SKOV-3-AA vs. SKOV-3-EV and SKOV-3-AA vs. SKOV-3-DD Cells with the Survival of Cisplatin-Treated Patients in The Cancer Genome Atlas (TCGA)

Next, we selected genes, which were differentially regulated in comparisons: SKOV-3-AA vs. SKOV-3-EV and SKOV-3-AA vs. SKOV-3-DD. Then we checked the expression levels of those 13 genes in the tumours of cisplatin-treated cancer patients and correlated them to patient survival (Table 2).

**Figure 4:**
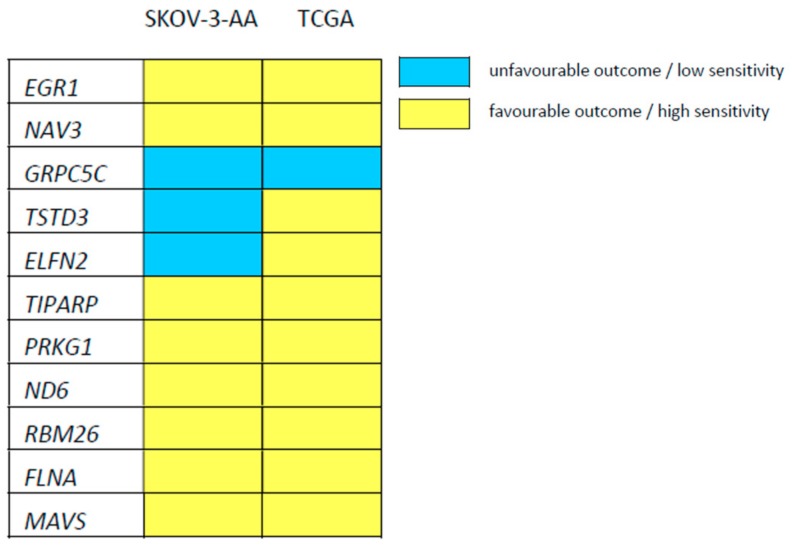
Heatmap indicating the relationship between low expression of the indicated genes and sensitivity/outcome, favourable (low cisplatin EC_50_ in SKOV-3-AA cells or prolonged survival of cisplatin-treated patients, indicated in yellow) or unfavourable (high cisplatin EC_50_ in SKOV-3-AA cells or reduced survival of cisplatin-treated patients, indicated in blue), based on the comparison of gene expression between SKOV-3-AA and EV- or PEA-15DD-transfected variants and TCGA data.

Patient survival is expressed as the hazard ratio at low (below median) expression of the respective gene in tumour tissue. A hazard ratio >1 means that the overall survival is reduced in patients with tumours that display low expression of the respective gene. A hazard ratio <1 indicates prolonged survival in patients, whose tumours display low expression of the respective genes. When we prepared a heatmap, in which we directly compared the effect of low expression of the investigated genes on cisplatin sensitivity or patient survival, there was substantial overlap as illustrated in Figure 4. The expression levels of nine of the eleven genes, for which gene expression data was available in the TCGA, were associated with beneficial (low EC_50_, prolonged survival) or poor (high EC_50_, shorter survival) outcome in the same way in both datasets (Table 2, Figure 4). There was a significant correlation (at a FDR of 0.2) for seven of these nine genes between their expression level in tumour tissue and patient survival: early growth response protein 1 (*EGR1*), neuron navigator 3 (*NAV3*), G Protein-Coupled Receptor Class C Group 5 Member C (*GPRC5C*), TCDD-inducible poly [ADP-ribose] polymerase (*TIPARP*), cGMP-dependent protein kinase 1 (*PRKG1*), filamin A (*FLNA*), mitochondrial antiviral-signalling protein (*MAVS*).

### 3.5. Pathway Analysis for the Genes Exclusively Regulated in SKOV-3-AA Cells Following Cisplatin Treatment

**Figure 5:**
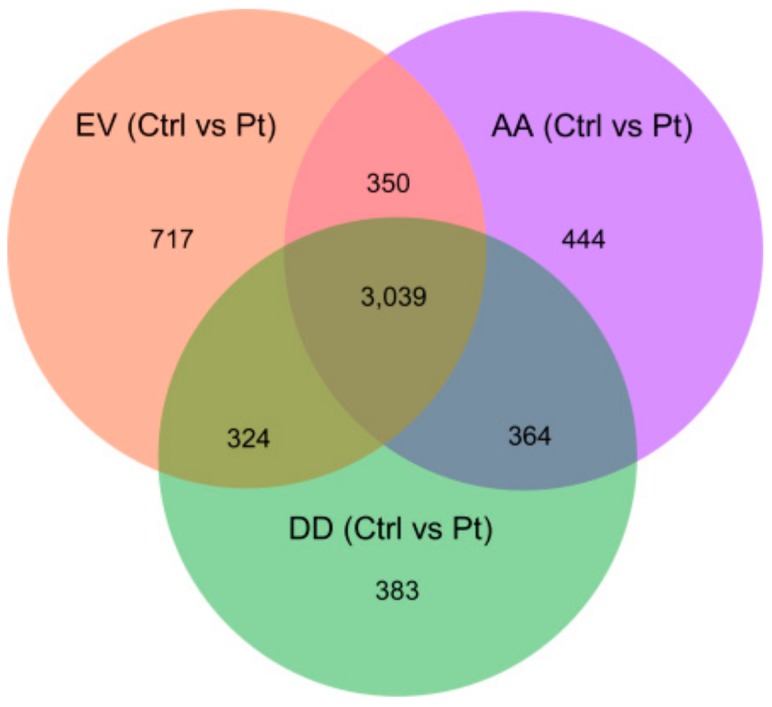
Venn diagram representing the exclusively and commonly regulated genes in different transfected cells upon cisplatin exposure. The diagram shows the total number of genes affected by cisplatin exposure in empty vector—(EV), PEA-15AA—(AA) and PEA-15DD-transfected—(DD) cells.

**Figure 6:**
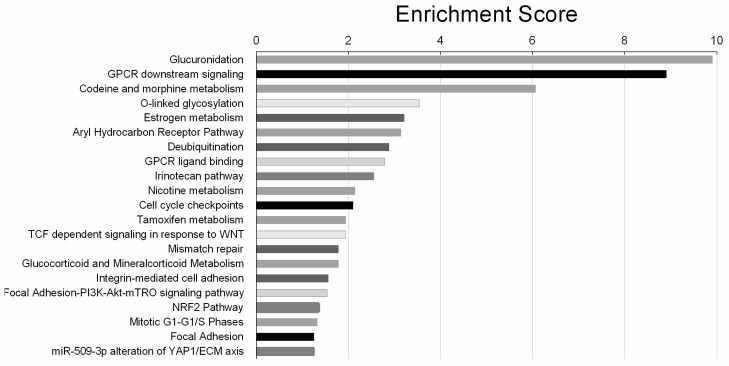
Twenty-one biological pathways significantly affected by cisplatin treatment in SKOV-3-AA cells, listed according to the significance level (log 2 base) in a descending order.

Since cisplatin treatment had a major impact on gene expression, we created a Venn diagram to define the genes exclusively regulated upon cisplatin treatment in different transfected cells. For all three cell types, 3039 differentially regulated genes were common. However, 717 genes were exclusively regulated after cisplatin exposure in SKOV-3-EV cells, while in SKOV-3-AA and SKOV-3-DD cells 444 genes and 383 genes were exclusively differentially expressed, respectively (Figure 5). The 444 genes that were found to be exclusively regulated in SKOV-3-AA cells upon cisplatin exposure may contribute to the increased cisplatin sensitivity in these cells. A pathway analysis based on Wikipathways revealed 21 pathways to be significantly affected in response to cisplatin treatment in SKOV-3-AA cells (Figure 6).

The most significantly affected pathway was the glucuronidation pathway with a significance value of 9.9 (log 2 base). Glucuronidation is the process of metabolizing substances such as drugs, pollutants, bilirubin, androgens, estrogens, glucocorticoids, fatty acids and bile acids. In glucuronidation process, the glucuronic acid of a uridine diphosphate (UDP) glucuronic acid is transferred to a substrate by UDP-glucuronyl transferase (UGT). The resulting substrates, glucuronides are more soluble in water and are excreted from body with urine and faeces. Among the other significant pathways, the Nrf2 pathway is of importance, as it is an upstream regulator of the UGTs. In addition, this pathway was previously found to influence cisplatin sensitivity in cancer cells [44].

### 3.6. Evaluation of the Responsible Genes Within the Affected Pathways

#### 3.6.1. UGT1A and Nrf2 Pathway

**Figure 7:**
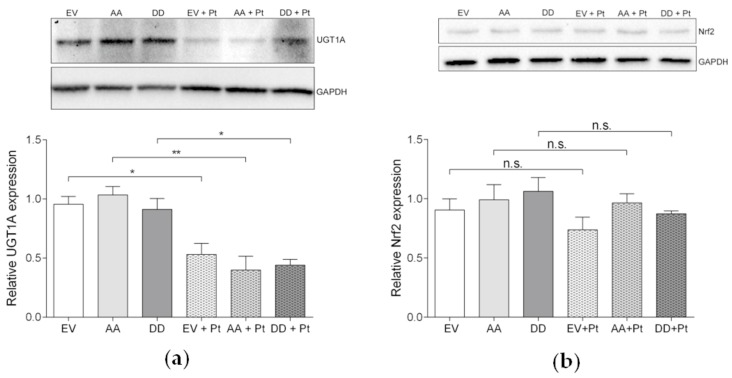
Representative Western blots and the corresponding densitometric quantification (mean ± SEM, *n* = 3) of (**a**) the relative uridine diphosphate-glucuronyl transferase (UGT)1A expression and (**b**) the relative nuclear factor erythroid 2-related factor 2 (Nrf2) expression in empty vector—(EV), PEA-15AA—(AA) and PEA-15DD-transfected—(DD) cells after treatment with 15 µM cisplatin (+Pt) for 24 h and in untreated transfected SKOV-3 cells. GAPDH was used as a loading control. * *p* < 0.05, ** *p* < 0.01.

All ten *UGT1A* isoforms were downregulated in SKOV-3-AA cells after cisplatin exposure according to the microarray data. Western blot analysis indicated that the expression of UGT1A protein in EV-, PEA-15AA- and PEA-15DD-transfected cells decreased in response to cisplatin to 55.63% (*p* = 0.02), 38.65% (*p* = 0.0013) and 48.27% (*p* = 0.01) of their basal level in untreated cells, respectively (Figure 7a). The greatest extent of reduction after cisplatin exposure was thus observed after PEA-15AA transfection (61.35% in SKOV-3-AA compared to 44.37% for SKOV-3-EV and 51.73% for SKOV-3-DD, along with much lower *p*-value).

The *UGT1A* family is also annotated in other pathways significantly affected by cisplatin treatment in SKOV-3-AA cells, including codeine and morphine metabolism, estrogen metabolism, aryl hydrocarbon receptor pathway, irinotecan pathway, nicotine metabolism, tamoxifen metabolism and Nrf2 pathway. Nrf2 is the upstream regulator of UGT1A expression. The array did not show any differential regulation of *Nrf2*, but some downstream genes of Nrf2 were differentially expressed. Therefore, the expression of Nrf2 was analysed on the protein level by Western blot in transfected cells before and after exposure to cisplatin (Figure 7b). The results showed a slight decrease in the overall Nrf2 levels after cisplatin exposure in all investigated cells. However, the differences were not statistically significant conforming to the result of the array.

#### 3.6.2. Retinoic Acid, an Inhibitor of Nrf2/ARE Pathway, Increases Cisplatin Sensitivity

Since UGT1A expression was affected by cisplatin in SKOV-3-AA cells, while Nrf2 expression was not, we hypothesised that Nrf2-associated downstream signalling via the Nrf2/ARE pathway may contribute to the sensitisation of PEA-15AA-transfected cells to cisplatin. All-trans retinoic acid (further retinoic acid), which is known to reduce the Nrf2-mediated induction of ARE-driven genes [45], was used to investigate this phenomenon further.

The EC_50_ value of retinoic acid was 216 µM (pEC_50_ = 3.660 ± 0.003, mean ± SEM, *n* = 6) after 48 h of incubation. In order to investigate the influence of the compound on cisplatin sensitivity, 20 µM of retinoic acid was used in combination with cisplatin in SKOV-3 cells as this concentration was lower than the EC_10_ of retinoic acid and therefore not toxic to the cells.

**Figure 8:**
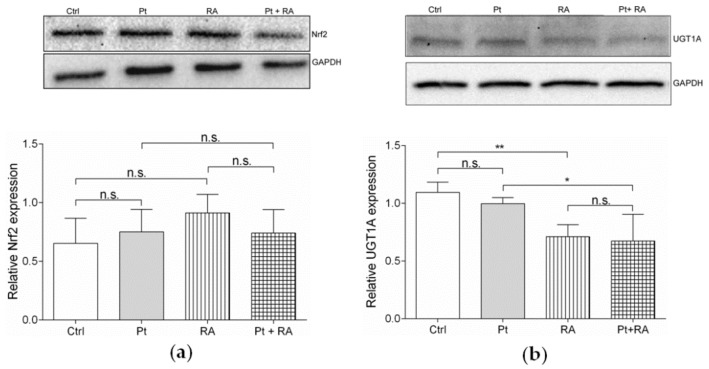
Representative Western blots and the corresponding densitometric quantification (mean ± SEM, *n* = 3) of (**a**) the relative Nrf2 expression and (**b**) the relative UGT1A expression in the transfected untreated SKOV-3 cells (Ctrl), after exposure to 15 µM cisplatin (Pt), to 20 µM retinoic acid (RA) and after co-incubation with 20 µM retinoic acid and 15 µM cisplatin (Pt + RA) for 24 h are shown. GAPDH was used as a loading control. *p < 0.05, ** *p* < 0.01, n.s. = not significant.

**Figure 9:**
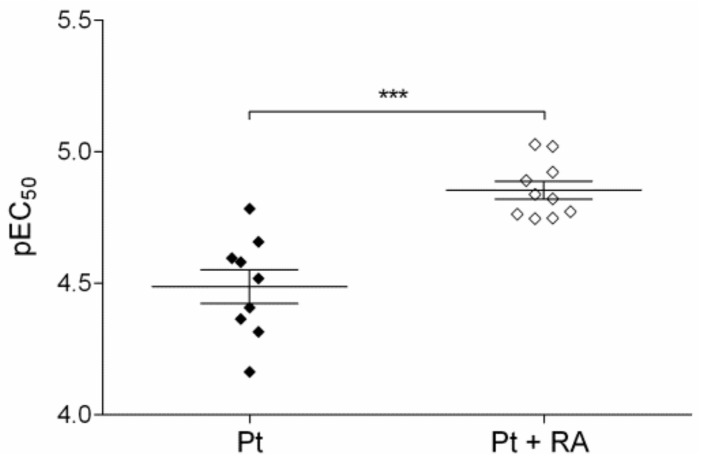
Sensitivity of SKOV-3 cells (pEC_50_, mean ± SEM, *n* = 9–10) of cisplatin alone (Pt), and upon co-incubation with 20 µM retinoic acid (Pt + RA) was determined over 48 h. *** *p* < 0.001.

Retinoic acid did not affect Nrf2 protein levels in all investigated cells independent of cisplatin treatment (Figure 8a). However, retinoic acid significantly reduced UGT1A levels alone and in combination with cisplatin (Figure 8b). This suggests that retinoic acid interferes with UGT1A expression via effects on Nrf2 signalling (but without directly affecting Nrf2 levels) similarly to PEA-15AA overexpression. In agreement, retinoic acid also significantly sensitised SKOV-3 cells to cisplatin (Figure 9). The EC_50_ value for cisplatin decreased from 32.6 µM to 13.9 µM in the presence of 20 µM retinoic acid. It should be noted that this experiment was conducted with non-transfected SKOV-3 cells, which cannot be directly compared with empty vector-transfected cells. Generally, after transfection process, cells became more sensitive to cisplatin. A similar effect of transfection procedure was also noticed in EFO27^r^CDDP^2000^ cells (Figure S1).

## 4. Discussion

Our results show that overexpression of PEA-15AA, a PEA-15 variant that cannot be phosphorylated at Ser104 and Ser116, increases the sensitivity of SKOV-3 ovarian cancer cells to cisplatin. The modest absolute effect of PEA-15 phosphorylation status on pEC_50_ may result from counteracting signalling pathways. Nevertheless, the finding was confirmed in an additional cell line model.

Previously, PEA-15 overexpression was found to induce cisplatin resistance and knockdown of PEA-15 was shown to enhance sensitivity to cisplatin in colorectal carcinoma cell line [46]. In that case, the wild-type PEA-15 was overexpressed, with both regulatory serine residues (Ser104 and Ser116) open to be phosphorylated. Therefore, the observed effects cannot be attributed to a single factor, i.e., overexpression of PEA-15. Our results indicate that PEA-15 phosphorylation status critically determines the cancer cell response to cisplatin. Non-phosphorylatable PEA-15AA had been previously found to inhibit ovarian cancer tumourigenicity and progression by blocking beta-catenin [33]. However, we did not observe direct effects of PEA-15AA on cell growth kinetics. PEA-15 phosphorylation at Ser116 rendered glioblastoma cells resistant to glucose-deprivation mediated cell death [47], which is in line with our finding that unphosphorylatable PEA-15 can potentiate cytotoxic effects. However, it appears to be not the case for all kinds of toxic stimuli. Interestingly, PEA-15AA had been shown to reduce ovarian cancer cell sensitivity to paclitaxel [34], a drug that interferes with microtubule disassembly [48], whereas cisplatin acts in a different fashion directly inducing DNA damage [1]. PEA-15 phosphorylation status affects several signalling pathways and apparently, depending on the mode of action, different downstream events gain more importance accounting for the diverging effects on cell sensitivity to different drugs.

In agreement with previous reports, PEA-15AA retained activated ERK1/2 in the cytoplasm. However, due to the diverse cellular functions of PEA-15, a microarray analysis was warranted to investigate the underlying key players of sensitisation of SKOV-3 cells to cisplatin by PEA-15AA.

In untreated cells, PEA-15-AA overexpression resulted in the differential regulation of thirteen genes between SKOV-3-AA and SKOV-3-EV or SKOV-3-DD cells. For eleven of these genes, data from cisplatin-treated patients was available in TCGA, and the expression of seven genes significantly correlated with the outcome of cisplatin therapy in the same way as expected from our data. Some of these genes are of particular interest in the context of ERK1/2 signalling. Early growth response protein 1 (*EGR1*) is a downstream nuclear target of Elk-1, which itself is regulated by activated ERK1/2 upon nuclear translocation [49,50], suggesting that PEA-15AA-mediated retention of activated ERK1/2 in the cytosol results in reduced *EGR1* expression. Low EGR1 levels have also been reported to correlate with high cisplatin sensitivity in primary glioma cells [51]. *FLNA*, coding for filamin A, a non-muscle actin filament cross-linking protein, is a downstream target of the nuclear transcription factor Sp1 [52], which is also regulated by ERK1/2 [53]. Reduced *FLNA* levels were observed in PEA-15AA-transfected cells in this study, and also correlated with increased cisplatin sensitivity in a mouse xenograft model [54] and with improved overall survival in patients [55] according to previous reports. Filamin A was also found to promote cancer growth [56] and to play an important role in repair of DNA damage [57].

A total of 444 genes were exclusively differentially regulated in cisplatin-treated SKOV-3-AA cells with UGT1A family representing the most significantly affected pathway (glucuronidation). UGTs catalyse the addition of a β-glucuronic acid moiety to a variety of nucleophilic sites of xenobiotics and endogenous compounds including bilirubin, steroids, bile acids, drugs and other carcinogenic and toxic compounds [58], and have been linked to drug resistance in cancer. The downregulation of UGT1A enzymes upon cisplatin exposure is likely to be a consequence of suppression of upstream signalling via Nrf2 pathway, which was also affected in response to cisplatin treatment. However, effects did not involve Nrf2 itself but seemed to be rather mediated by downstream signalling events. Retinoic acid, a known inhibitor of Nrf2/ARE signalling reduced UGT1A levels and increased cisplatin sensitivity of SKOV-3 cells independently of Nrf2 expression in a similar fashion as PEA-15AA overexpression.

PEA-15 was already evaluated for treating advanced breast cancer in mice. To target PEA-15 in advanced breast tumours, Xie et al. developed a breast cancer specific construct (T-VISA) composed of the human telomerase reverse transcriptase (hTERT; T) promoter and a versatile transgene amplification vector VISA (VP16-GAL4-WPRE integrated systemic amplifier). T-VISA-PEA-15 was found to be highly specific, to selectively express PEA-15 in breast cancer cells, and to induce cancer cell killing in vitro and in vivo without affecting normal cells [58]. A similar construct with PEA-15AA would be of interest for ovarian cancer in order to investigate if the efficacy of cisplatin treatment can be improved.

In conclusion, non-phosphorylatable PEA-15AA increases cisplatin sensitivity of ovarian cancer cells, suggesting that the phosphorylation status of PEA-15 could be evaluated as a biomarker to predict the responsiveness of ovarian tumours to cisplatin treatment. For this purpose, correlation of PEA-15 phosphorylation with therapy outcome in high-grade serous ovarian cancer is needed, as this subtype is the most aggressive and has the worst prognosis [59].

## Figures and Tables

**Table 1:** Number of differentially expressed genes, compared as treatment condition 1 vs. condition 2 with at least two-fold up- or down-regulation with *p* < 0.05.

Treatment Condition 1	Treatment Condition 2	Number of Genes
SKOV-3-EV, untreated	SKOV-3- AA, untreated	3
SKOV-3-EV, untreated	SKOV-3-DD, untreated	18
SKOV-3-AA, untreated	SKOV-3- DD, untreated	10
SKOV-3-EV, untreated	SKOV-3-EV, 15 µM cisplatin, 24 h	4430
SKOV-3-AA, untreated	SKOV-3-AA, 15 µM cisplatin, 24 h	4197
SKOV-3-DD, untreated	SKOV-3-DD, 15 µM cisplatin, 24 h	4110

**Table 2:** Correlation of the expression of genes, differentially expressed between SKOV-3-AA and SKOV-3-EV or SKOV-3-DD cells, in tumours of cisplatin-treated patients (*n* = 779) and patient survival.

Gene	Fold Change	Hazard Ratio ^1^	*p*-Value	FDR (thr. 0.2) ^2^	FDR (thr. 0.05)
*EGR1*	−2.22	0.7068	0.00322 **	0.034099	0.008525
*NAV3*	−2.18	0.7062	0.00317 **	0.033948	0.008487
*GPRC5C*	2.14	1.4745	0.000971 **	0.023936	0.005984
**Gene**	**Fold change**	**Hazard ratio ^1^**	***p*-value**	**FDR (thr. 0.2) ^2^**	**FDR (thr. 0.05)**
*TSTD3*	2.16	0.7545	0.0162 *	0.053933	0.013483
*ELFN2*	2.09	0.9036	0.385	0.139261	0.034815
*LOC100287225*	−2.37	n.a. ^3^	n.a.	n.a.	n.a.
*TIPARP*	−2.19	0.7505	0.015 *	0.052694	0.013174
*PRKG1*	−2.00	0.7532	0.0154 *	0.053205	0.013301
*ND6*	−2.07	0.8492	0.163	0.10641	0.026602
*RBM26*	−2.42	0.8951	0.342	0.133674	0.033418
*FLNA*	−2.86	0.7523	0.0152 *	0.052997	0.013249
*MAVS*	−2.24	0.6656	0.000569 **	0.020231	0.005058
*GTSF1L*	−2.07	n.a.	n.a.	n.a.	n.a.

^1^ Hazard ratio at low gene expression levels in tumour tissue; ^2^ thr. = false discovery rate threshold determined according to Benjamini-Hochberg [43]; ^3^ n.a. = not applicable; * = *p*-value below 0.05 and significant at FDR = 0.2; ** = *p*-value below 0.05 and significant at FDR = 0.05.

## Supplementary Materials

The following are available online at https://www.mdpi.com/2073-4409/9/2/515/s1, Figure S1: (**a**) Expression of hemagglutinin (HA)-tagged PEA-15 in EFO27^r^CDDP^2000^ cells after transfection with the HA-tagged empty vector (EV), PEA-15AA (AA) and PEA-15DD (DD). GAPDH was used as a loading control. (**b**) Cisplatin cytotoxicity (pEC_50_, mean ± SEM, *n* = 8) in non-transfected EFO27^r^CDDP^2000^ cells, cells transfected with empty vector (EV), with PEA-15AA (AA), and with PEA-15DD (DD). ***p < 0.001, n.s. = not significant.
