# Supplementary material for: Non-Phosphorylatable PEA-15 Sensitises SKOV-3 Ovarian Cancer Cells to Cisplatin
Source: Cells. 2020 Feb 24;9(2):515. doi: 10.3390/cells9020515 (PMC7072772; doi:10.3390/cells9020515)
Supplement: Supplementary file 1 [file cells-09-00515-s001.pdf]

Non-phosphorylatable PEA-15 sensitises ovarian cancer cells to cisplatin

Shahana Dilruba, Alessia Grondana, Anke C. Schiedel, Naoto T. Ueno, Chandra Bartholomeusz, Jindrich Cinatl jr., Katie-May McLaughlin, Mark N. Wass, Martin Michaelis, and Ganna V. Kalayda*

Supplementary Information

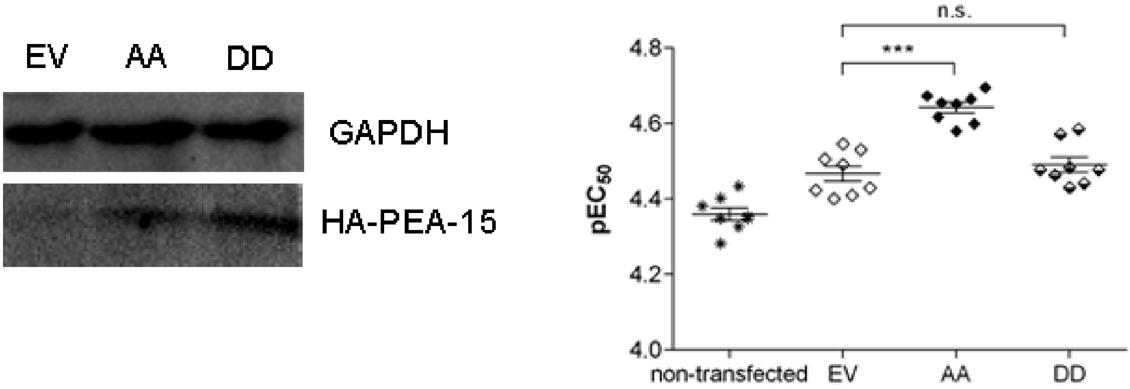

(a) (b)

Figure S1. (**a**) Expression of hemagglutinin (HA)-tagged PEA-15 in $EFO27^{r}CDDP^{2000}$ cells after transfection with the HA-tagged empty vector (EV), PEA-15AA (AA) and PEA-15DD (DD). GAPDH was used as a loading control. (**b**) Cisplatin cytotoxicity (pEC_{50}, mean ± SEM, n = 8) in non-transfected $EFO27^{r}CDDP^{2000}$ cells, cells transfected with empty vector (EV), with PEA-15AA (AA), and with PEA-15DD (DD). ***$p < 0.001$, n.s. = not significant.
